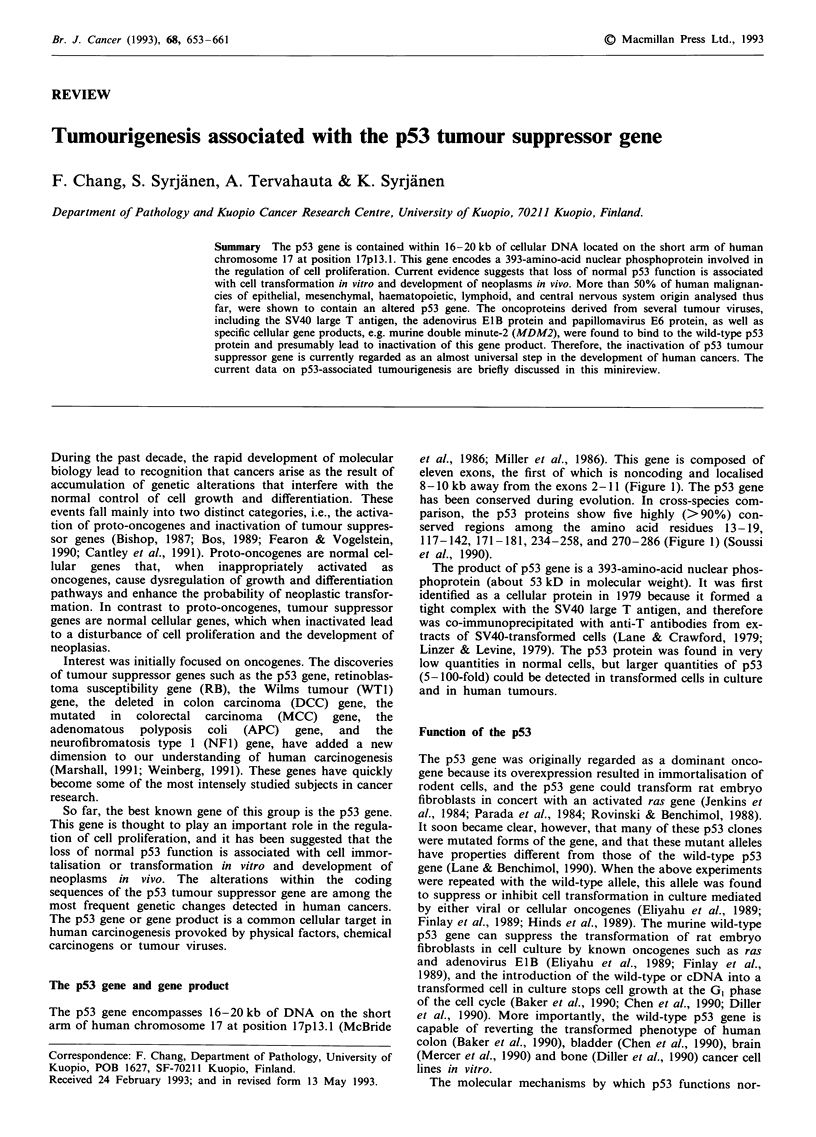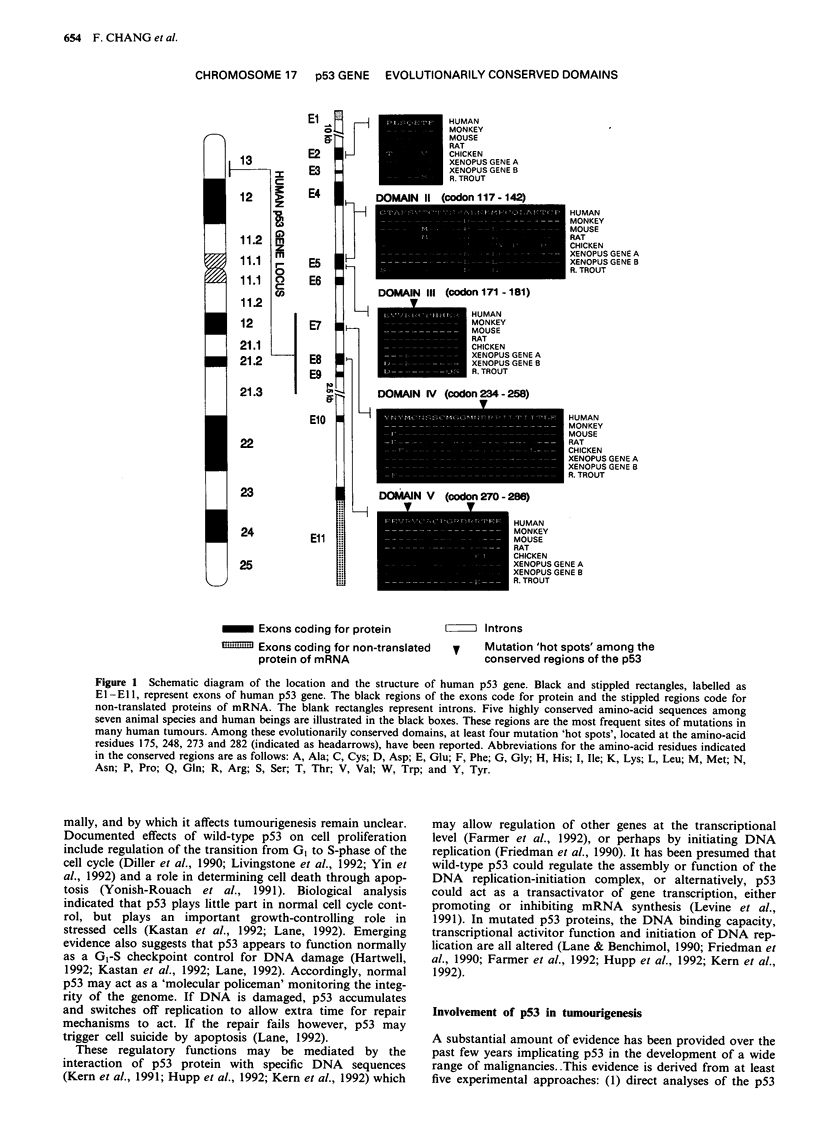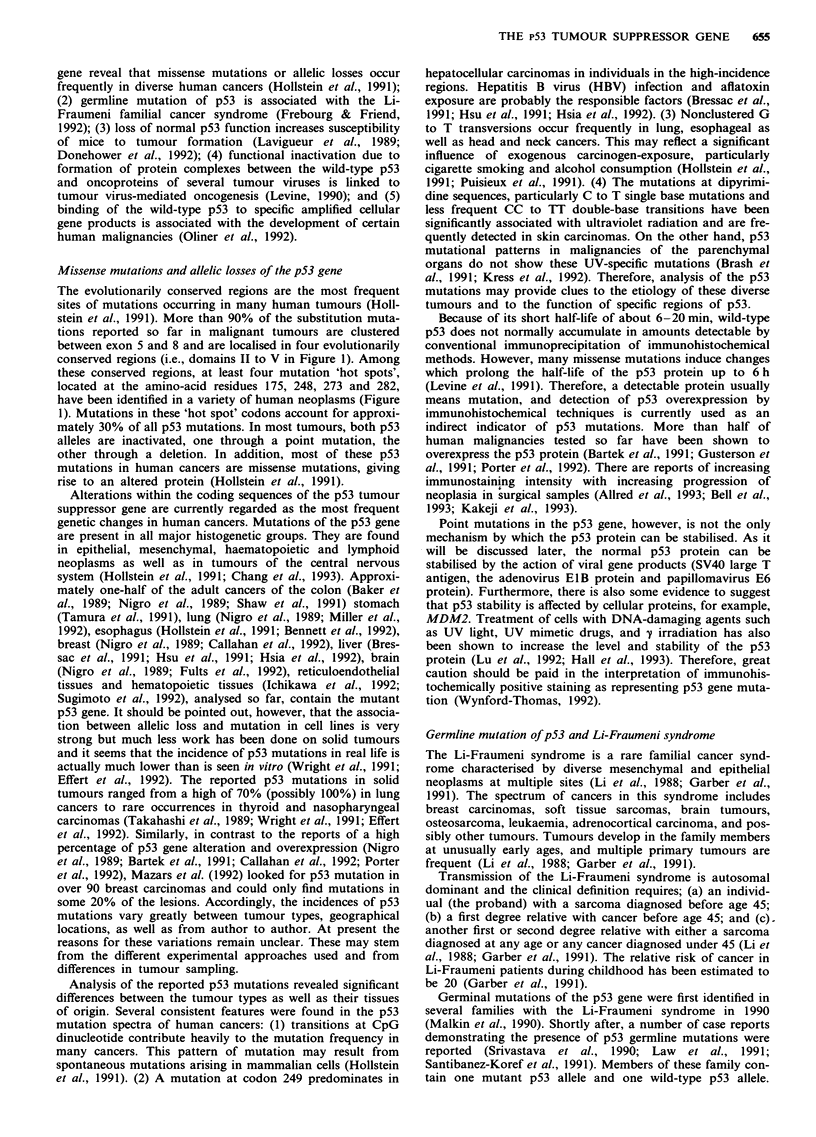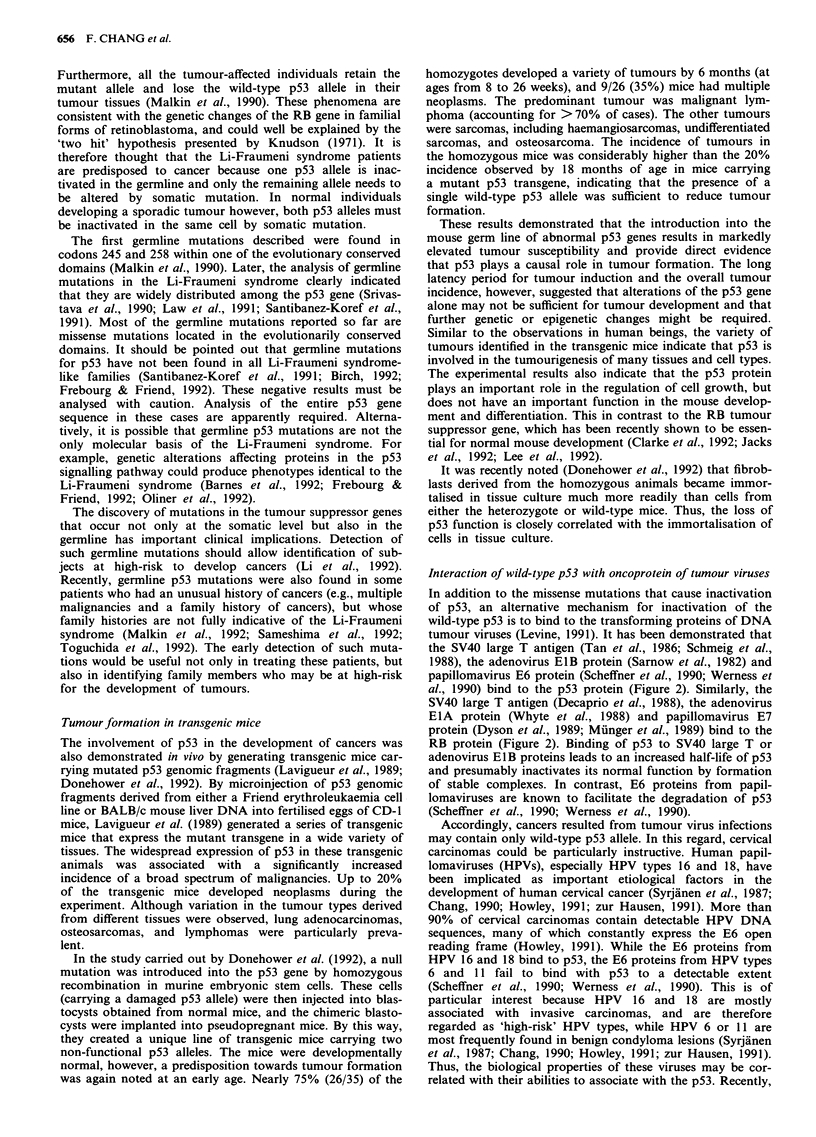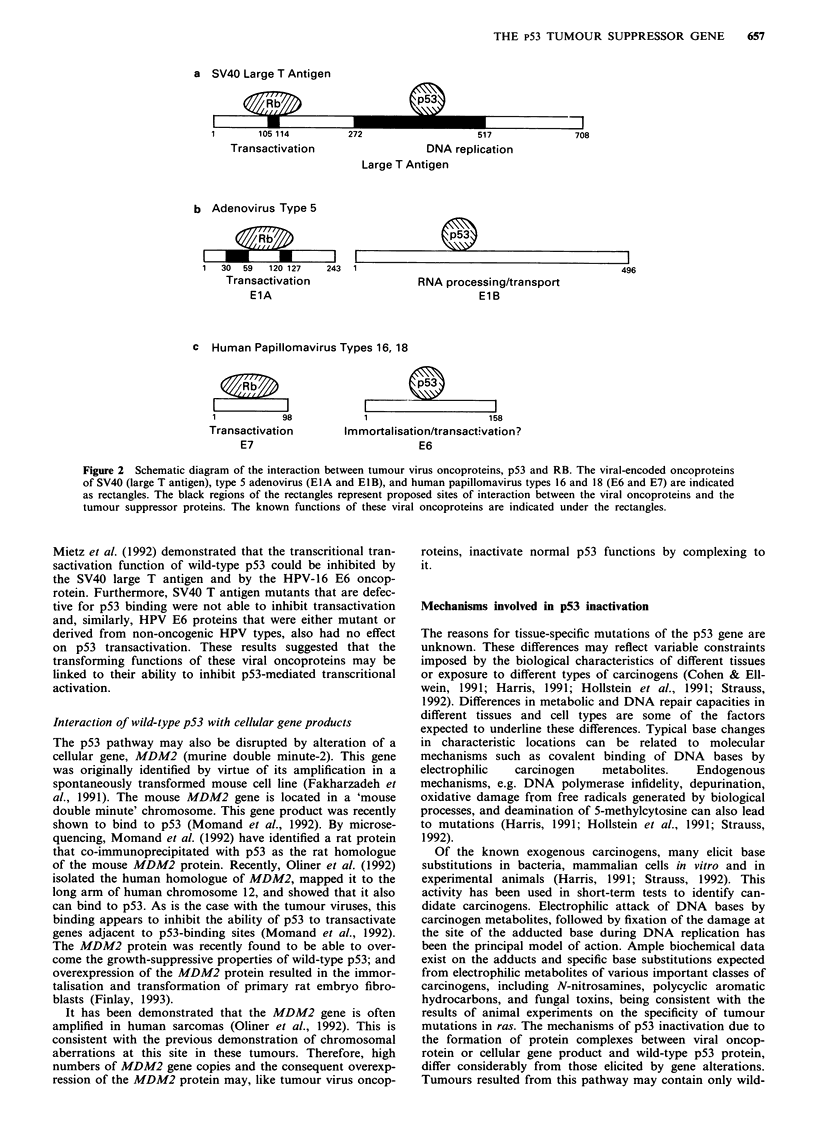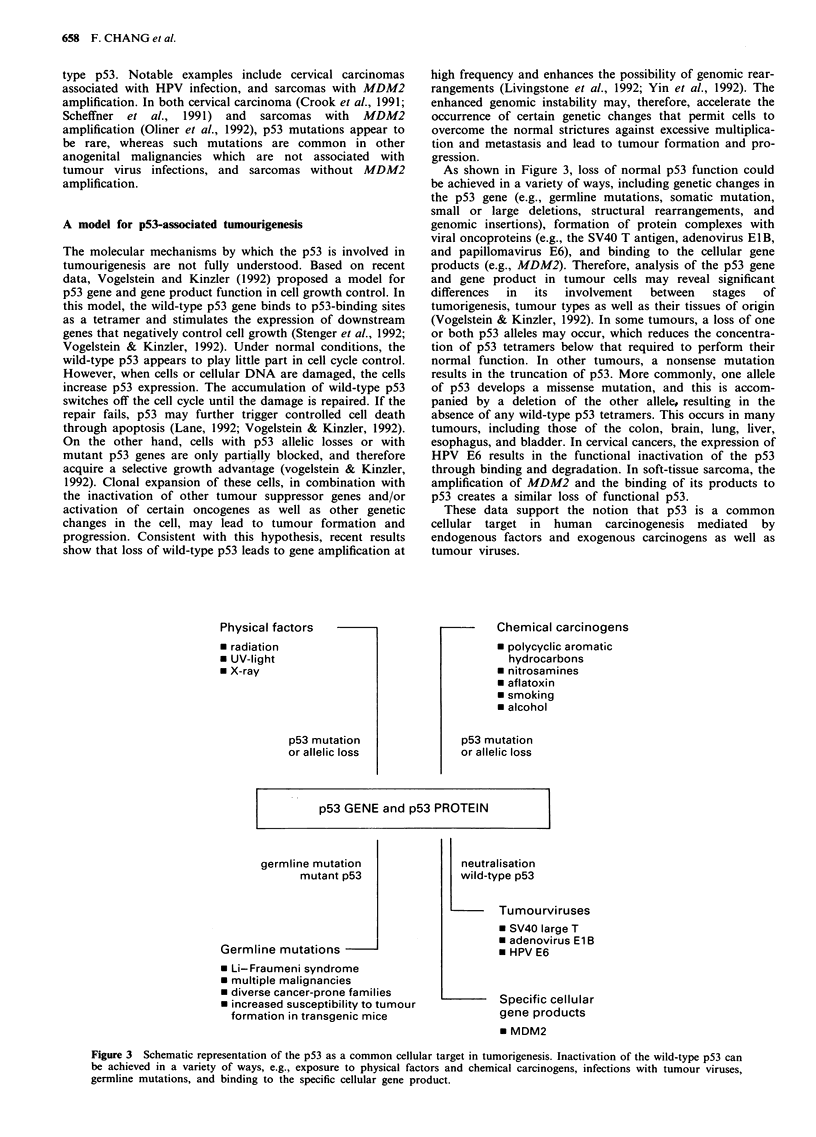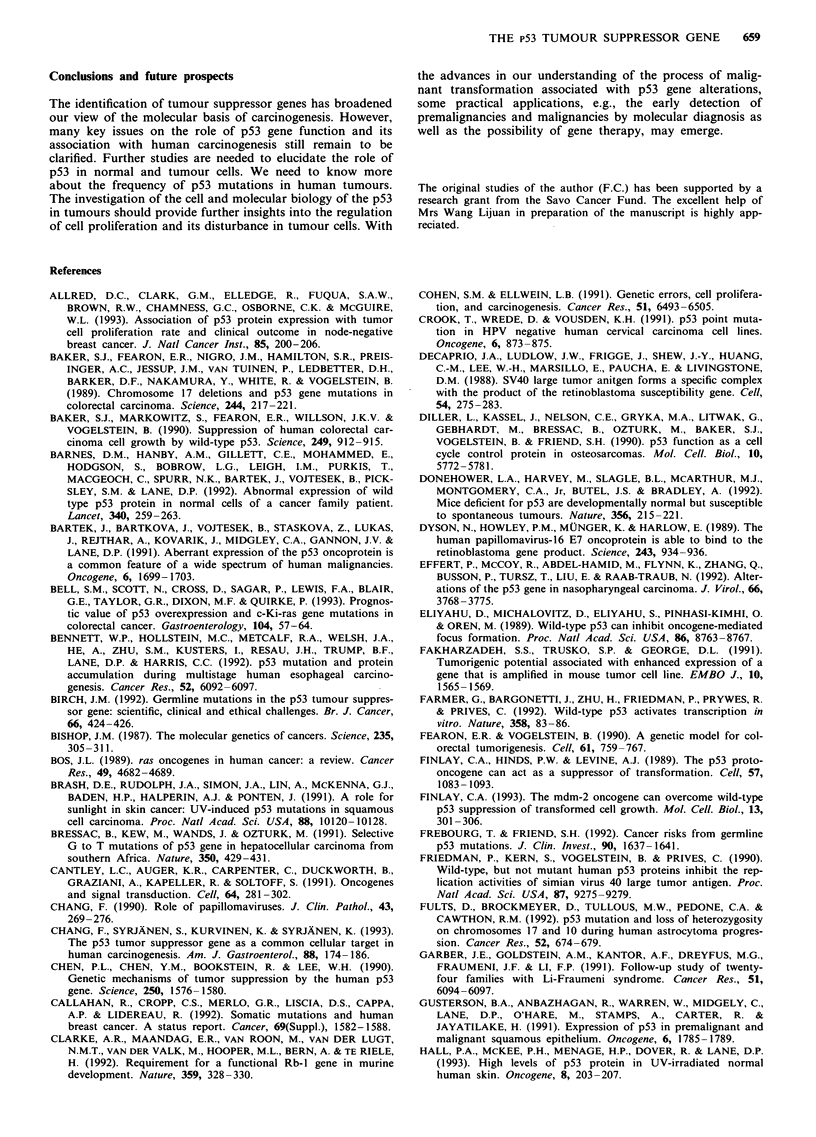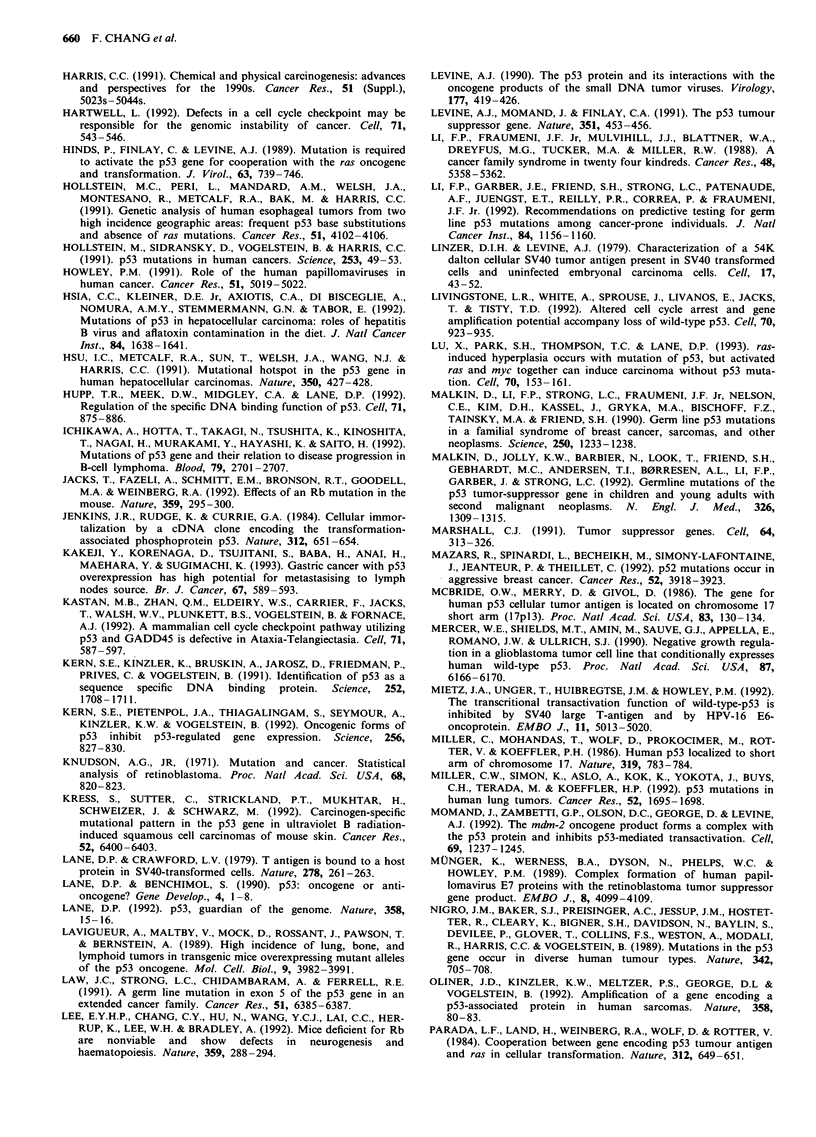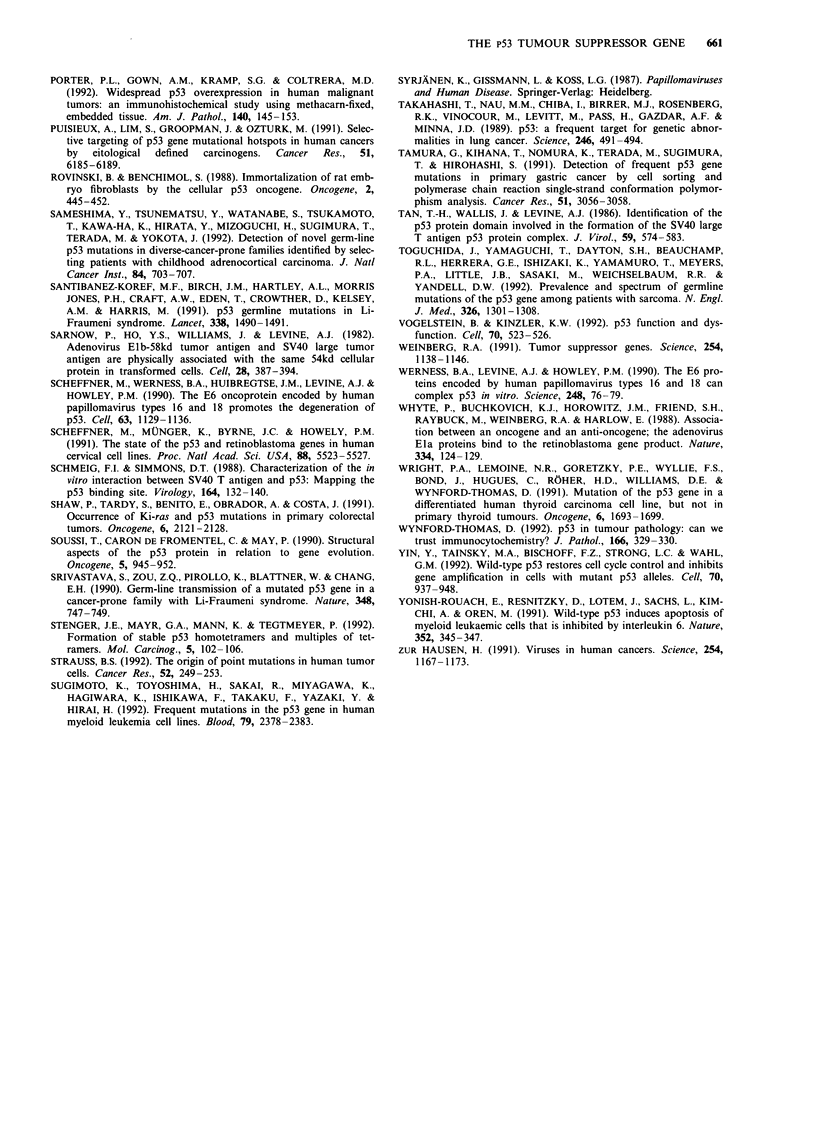# Tumourigenesis associated with the p53 tumour suppressor gene.

**DOI:** 10.1038/bjc.1993.404

**Published:** 1993-10

**Authors:** F. Chang, S. Syrjänen, A. Tervahauta, K. Syrjänen

**Affiliations:** Department of Pathology, University of Kuopio, Finland.

## Abstract

The p53 gene is contained within 16-20 kb of cellular DNA located on the short arm of human chromosome 17 at position 17p13.1. This gene encodes a 393-amino-acid nuclear phosphoprotein involved in the regulation of cell proliferation. Current evidence suggests that loss of normal p53 function is associated with cell transformation in vitro and development of neoplasms in vivo. More than 50% of human malignancies of epithelial, mesenchymal, haematopoietic, lymphoid, and central nervous system origin analysed thus far, were shown to contain an altered p53 gene. The oncoproteins derived from several tumour viruses, including the SV40 large T antigen, the adenovirus E1B protein and papillomavirus E6 protein, as well as specific cellular gene products, e.g. murine double minute-2 (MDM2), were found to bind to the wild-type p53 protein and presumably lead to inactivation of this gene product. Therefore, the inactivation of p53 tumour suppressor gene is currently regarded as an almost universal step in the development of human cancers. The current data on p53-associated tumourigenesis are briefly discussed in this minireview.


Br. J. Cancer (1993), 68, 653–661 © Macmillan Press Ltd., 1993

**REVIEW**

### Tumourigenesis associated with the p53 tumour suppressor gene

F. Chang, S. Syrjänen, A. Tervahauta & K. Syrjänen

*Department of Pathology and Kuopio Cancer Research Centre, University of Kuopio, 70211 Kuopio, Finland.*

**Summary** The p53 gene is contained within 16–20 kb of cellular DNA located on the short arm of human chromosome 17 at position 17p13.1. This gene encodes a 393-amino-acid nuclear phosphoprotein involved in the regulation of cell proliferation. Current evidence suggests that loss of normal p53 function is associated with cell transformation *in vitro* and development of neoplasms *in vivo*. More than 50% of human malignancies of epithelial, mesenchymal, haematopoietic, lymphoid, and central nervous system origin analysed thus far, were shown to contain an altered p53 gene. The oncoproteins derived from several tumour viruses, including the SV40 large T antigen, the adenovirus E1B protein and papillomavirus E6 protein, as well as specific cellular gene products, e.g. murine double minute-2 (*MDM2*), were found to bind to the wild-type p53 protein and presumably lead to inactivation of this gene product. Therefore, the inactivation of p53 tumour suppressor gene is currently regarded as an almost universal step in the development of human cancers. The current data on p53-associated tumourigenesis are briefly discussed in this minireview.

During the past decade, the rapid development of molecular biology lead to recognition that cancers arise as the result of accumulation of genetic alterations that interfere with the normal control of cell growth and differentiation. These events fall mainly into two distinct categories, i.e., the activation of proto-oncogenes and inactivation of tumour suppressor genes (Bishop, 1987; Bos, 1989; Fearon & Vogelstein, 1990; Cantley *et al.*, 1991). Proto-oncogenes are normal cellular genes that, when inappropriately activated as oncogenes, cause dysregulation of growth and differentiation pathways and enhance the probability of neoplastic transformation. In contrast to proto-oncogenes, tumour suppressor genes are normal cellular genes, which when inactivated lead to a disturbance of cell proliferation and the development of neoplasias.

Interest was initially focused on oncogenes. The discoveries of tumour suppressor genes such as the p53 gene, retinoblastoma susceptibility gene (RB), the Wilms tumour (WT1) gene, the deleted in colon carcinoma (DCC) gene, the mutated in colorectal carcinoma (MCC) gene, the adenomatous polyposis coli (APC) gene, and the neurofibromatosis type 1 (NF1) gene, have added a new dimension to our understanding of human carcinogenesis (Marshall, 1991; Weinberg, 1991). These genes have quickly become some of the most intensely studied subjects in cancer research.

So far, the best known gene of this group is the p53 gene. This gene is thought to play an important role in the regulation of cell proliferation, and it has been suggested that the loss of normal p53 function is associated with cell immortalisation or transformation *in vitro* and development of neoplasms *in vivo*. The alterations within the coding sequences of the p53 tumour suppressor gene are among the most frequent genetic changes detected in human cancers. The p53 gene or gene product is a common cellular target in human carcinogenesis provoked by physical factors, chemical carcinogens or tumour viruses.

### The p53 gene and gene product

The p53 gene encompasses 16–20 kb of DNA on the short arm of human chromosome 17 at position 17p13.1 (McBride *et al.*, 1986; Miller *et al.*, 1986). This gene is composed of eleven exons, the first of which is noncoding and localised 8–10 kb away from the exons 2–11 (Figure 1). The p53 gene has been conserved during evolution. In cross-species comparison, the p53 proteins show five highly (>90%) conserved regions among the amino acid residues 13–19, 117–142, 171–181, 234–258, and 270–286 (Figure 1) (Soussi *et al.*, 1990).

The product of p53 gene is a 393-amino-acid nuclear phosphoprotein (about 53 kD in molecular weight). It was first identified as a cellular protein in 1979 because it formed a tight complex with the SV40 large T antigen, and therefore was co-immunoprecipitated with anti-T antibodies from extracts of SV40-transformed cells (Lane & Crawford, 1979; Linzer & Levine, 1979). The p53 protein was found in very low quantities in normal cells, but larger quantities of p53 (5–100-fold) could be detected in transformed cells in culture and in human tumours.

### Function of the p53

The p53 gene was originally regarded as a dominant oncogene because its overexpression resulted in immortalisation of rodent cells, and the p53 gene could transform rat embryo fibroblasts in concert with an activated *ras* gene (Jenkins *et al.*, 1984; Parada *et al.*, 1984; Rovinski & Benchimol, 1988). It soon became clear, however, that many of these p53 clones were mutated forms of the gene, and that these mutant alleles have properties different from those of the wild-type p53 gene (Lane & Benchimol, 1990). When the above experiments were repeated with the wild-type allele, this allele was found to suppress or inhibit cell transformation in culture mediated by either viral or cellular oncogenes (Eliyahu *et al.*, 1989; Finlay *et al.*, 1989; Hinds *et al.*, 1989). The murine wild-type p53 gene can suppress the transformation of rat embryo fibroblasts in cell culture by known oncogenes such as *ras* and adenovirus E1B (Eliyahu *et al.*, 1989; Finlay *et al.*, 1989), and the introduction of the wild-type or cDNA into a transformed cell in culture stops cell growth at the $G_1$ phase of the cell cycle (Baker *et al.*, 1990; Chen *et al.*, 1990; Diller *et al.*, 1990). More importantly, the wild-type p53 gene is capable of reverting the transformed phenotype of human colon (Baker *et al.*, 1990), bladder (Chen *et al.*, 1990), brain (Mercer *et al.*, 1990) and bone (Diller *et al.*, 1990) cancer cell lines *in vitro*.

The molecular mechanisms by which p53 functions nor-

Correspondence: F. Chang, Department of Pathology, University of Kuopio, POB 1627, SF-70211 Kuopio, Finland.
Received 24 February 1993; and in revised form 13 May 1993.

**Figure 1** Schematic diagram of the location and the structure of human p53 gene. Black and stippled rectangles, labelled as E1–E11, represent exons of human p53 gene. The black regions of the exons code for protein and the stippled regions code for non-translated proteins of mRNA. The blank rectangles represent introns. Five highly conserved amino-acid sequences among seven animal species and human beings are illustrated in the black boxes. These regions are the most frequent sites of mutations in many human tumours. Among these evolutionarily conserved domains, at least four mutation 'hot spots', located at the amino-acid residues 175, 248, 273 and 282 (indicated as headarrows), have been reported. Abbreviations for the amino-acid residues indicated in the conserved regions are as follows: A, Ala; C, Cys; D, Asp; E, Glu; F, Phe; G, Gly; H, His; I, Ile; K, Lys; L, Leu; M, Met; N, Asn; P, Pro; Q, Gln; R, Arg; S, Ser; T, Thr; V, Val; W, Trp; and Y, Tyr.

mally, and by which it affects tumourigenesis remain unclear. Documented effects of wild-type p53 on cell proliferation include regulation of the transition from $G_1$ to S-phase of the cell cycle (Diller *et al.*, 1990; Livingstone *et al.*, 1992; Yin *et al.*, 1992) and a role in determining cell death through apoptosis (Yonish-Rouach *et al.*, 1991). Biological analysis indicated that p53 plays little part in normal cell cycle control, but plays an important growth-controlling role in stressed cells (Kastan *et al.*, 1992; Lane, 1992). Emerging evidence also suggests that p53 appears to function normally as a $G_1$-S checkpoint control for DNA damage (Hartwell, 1992; Kastan *et al.*, 1992; Lane, 1992). Accordingly, normal p53 may act as a 'molecular policeman' monitoring the integrity of the genome. If DNA is damaged, p53 accumulates and switches off replication to allow extra time for repair mechanisms to act. If the repair fails however, p53 may trigger cell suicide by apoptosis (Lane, 1992).

These regulatory functions may be mediated by the interaction of p53 protein with specific DNA sequences (Kern *et al.*, 1991; Hupp *et al.*, 1992; Kern *et al.*, 1992) which may allow regulation of other genes at the transcriptional level (Farmer *et al.*, 1992), or perhaps by initiating DNA replication (Friedman *et al.*, 1990). It has been presumed that wild-type p53 could regulate the assembly or function of the DNA replication-initiation complex, or alternatively, p53 could act as a transactivator of gene transcription, either promoting or inhibiting mRNA synthesis (Levine *et al.*, 1991). In mutated p53 proteins, the DNA binding capacity, transcriptional activitor function and initiation of DNA replication are all altered (Lane & Benchimol, 1990; Friedman *et al.*, 1990; Farmer *et al.*, 1992; Hupp *et al.*, 1992; Kern *et al.*, 1992).

**Involvement of p53 in tumourigenesis**

A substantial amount of evidence has been provided over the past few years implicating p53 in the development of a wide range of malignancies. .This evidence is derived from at least five experimental approaches: (1) direct analyses of the p53

gene reveal that missense mutations or allelic losses occur frequently in diverse human cancers (Hollstein *et al.*, 1991); (2) germline mutation of p53 is associated with the Li-Fraumeni familial cancer syndrome (Frebourg & Friend, 1992); (3) loss of normal p53 function increases susceptibility of mice to tumour formation (Lavigueur *et al.*, 1989; Donehower *et al.*, 1992); (4) functional inactivation due to formation of protein complexes between the wild-type p53 and oncoproteins of several tumour viruses is linked to tumour virus-mediated oncogenesis (Levine, 1990); and (5) binding of the wild-type p53 to specific amplified cellular gene products is associated with the development of certain human malignancies (Oliner *et al.*, 1992).

*Missense mutations and allelic losses of the p53 gene*

The evolutionarily conserved regions are the most frequent sites of mutations occurring in many human tumours (Hollstein *et al.*, 1991). More than 90% of the substitution mutations reported so far in malignant tumours are clustered between exon 5 and 8 and are localised in four evolutionarily conserved regions (i.e., domains II to V in Figure 1). Among these conserved regions, at least four mutation 'hot spots', located at the amino-acid residues 175, 248, 273 and 282, have been identified in a variety of human neoplasms (Figure 1). Mutations in these 'hot spot' codons account for approximately 30% of all p53 mutations. In most tumours, both p53 alleles are inactivated, one through a point mutation, the other through a deletion. In addition, most of these p53 mutations in human cancers are missense mutations, giving rise to an altered protein (Hollstein *et al.*, 1991).

Alterations within the coding sequences of the p53 tumour suppressor gene are currently regarded as the most frequent genetic changes in human cancers. Mutations of the p53 gene are present in all major histogenetic groups. They are found in epithelial, mesenchymal, haematopoietic and lymphoid neoplasms as well as in tumours of the central nervous system (Hollstein *et al.*, 1991; Chang *et al.*, 1993). Approximately one-half of the adult cancers of the colon (Baker *et al.*, 1989; Nigro *et al.*, 1989; Shaw *et al.*, 1991) stomach (Tamura *et al.*, 1991), lung (Nigro *et al.*, 1989; Miller *et al.*, 1992), esophagus (Hollstein *et al.*, 1991; Bennett *et al.*, 1992), breast (Nigro *et al.*, 1989; Callahan *et al.*, 1992), liver (Bressac *et al.*, 1991; Hsu *et al.*, 1991; Hsia *et al.*, 1992), brain (Nigro *et al.*, 1989; Fults *et al.*, 1992), reticuloendothelial tissues and hematopoietic tissues (Ichikawa *et al.*, 1992; Sugimoto *et al.*, 1992), analysed so far, contain the mutant p53 gene. It should be pointed out, however, that the association between allelic loss and mutation in cell lines is very strong but much less work has been done on solid tumours and it seems that the incidence of p53 mutations in real life is actually much lower than is seen *in vitro* (Wright *et al.*, 1991; Effert *et al.*, 1992). The reported p53 mutations in solid tumours ranged from a high of 70% (possibly 100%) in lung cancers to rare occurrences in thyroid and nasopharyngeal carcinomas (Takahashi *et al.*, 1989; Wright *et al.*, 1991; Effert *et al.*, 1992). Similarly, in contrast to the reports of a high percentage of p53 gene alteration and overexpression (Nigro *et al.*, 1989; Bartek *et al.*, 1991; Callahan *et al.*, 1992; Porter *et al.*, 1992), Mazars et al. (1992) looked for p53 mutation in over 90 breast carcinomas and could only find mutations in some 20% of the lesions. Accordingly, the incidences of p53 mutations vary greatly between tumour types, geographical locations, as well as from author to author. At present the reasons for these variations remain unclear. These may stem from the different experimental approaches used and from differences in tumour sampling.

Analysis of the reported p53 mutations revealed significant differences between the tumour types as well as their tissues of origin. Several consistent features were found in the p53 mutation spectra of human cancers: (1) transitions at CpG dinucleotide contribute heavily to the mutation frequency in many cancers. This pattern of mutation may result from spontaneous mutations arising in mammalian cells (Hollstein *et al.*, 1991). (2) A mutation at codon 249 predominates in hepatocellular carcinomas in individuals in the high-incidence regions. Hepatitis B virus (HBV) infection and aflatoxin exposure are probably the responsible factors (Bressac *et al.*, 1991; Hsu *et al.*, 1991; Hsia *et al.*, 1992). (3) Nonclustered G to T transversions occur frequently in lung, esophageal as well as head and neck cancers. This may reflect a significant influence of exogenous carcinogen-exposure, particularly cigarette smoking and alcohol consumption (Hollstein *et al.*, 1991; Puisieux *et al.*, 1991). (4) The mutations at dipyrimidine sequences, particularly C to T single base mutations and less frequent CC to TT double-base transitions have been significantly associated with ultraviolet radiation and are frequently detected in skin carcinomas. On the other hand, p53 mutational patterns in malignancies of the parenchymal organs do not show these UV-specific mutations (Brash *et al.*, 1991; Kress *et al.*, 1992). Therefore, analysis of the p53 mutations may provide clues to the etiology of these diverse tumours and to the function of specific regions of p53.

Because of its short half-life of about 6–20 min, wild-type p53 does not normally accumulate in amounts detectable by conventional immunoprecipitation of immunohistochemical methods. However, many missense mutations induce changes which prolong the half-life of the p53 protein up to 6 h (Levine *et al.*, 1991). Therefore, a detectable protein usually means mutation, and detection of p53 overexpression by immunohistochemical techniques is currently used as an indirect indicator of p53 mutations. More than half of human malignancies tested so far have been shown to overexpress the p53 protein (Bartek *et al.*, 1991; Gusterson *et al.*, 1991; Porter *et al.*, 1992). There are reports of increasing immunostaining intensity with increasing progression of neoplasia in surgical samples (Allred *et al.*, 1993; Bell *et al.*, 1993; Kakeji *et al.*, 1993).

Point mutations in the p53 gene, however, is not the only mechanism by which the p53 protein can be stabilised. As it will be discussed later, the normal p53 protein can be stabilised by the action of viral gene products (SV40 large T antigen, the adenovirus E1B protein and papillomavirus E6 protein). Furthermore, there is also some evidence to suggest that p53 stability is affected by cellular proteins, for example, *MDM2*. Treatment of cells with DNA-damaging agents such as UV light, UV mimetic drugs, and $\gamma$ irradiation has also been shown to increase the level and stability of the p53 protein (Lu *et al.*, 1992; Hall *et al.*, 1993). Therefore, great caution should be paid in the interpretation of immunohistochemically positive staining as representing p53 gene mutation (Wynford-Thomas, 1992).

*Germline mutation of p53 and Li-Fraumeni syndrome*

The Li-Fraumeni syndrome is a rare familial cancer syndrome characterised by diverse mesenchymal and epithelial neoplasms at multiple sites (Li *et al.*, 1988; Garber *et al.*, 1991). The spectrum of cancers in this syndrome includes breast carcinomas, soft tissue sarcomas, brain tumours, osteosarcoma, leukaemia, adrenocortical carcinoma, and possibly other tumours. Tumours develop in the family members at unusually early ages, and multiple primary tumours are frequent (Li *et al.*, 1988; Garber *et al.*, 1991).

Transmission of the Li-Fraumeni syndrome is autosomal dominant and the clinical definition requires; (a) an individual (the proband) with a sarcoma diagnosed before age 45; (b) a first degree relative with cancer before age 45; and (c) another first or second degree relative with either a sarcoma diagnosed at any age or any cancer diagnosed under 45 (Li *et al.*, 1988; Garber *et al.*, 1991). The relative risk of cancer in Li-Fraumeni patients during childhood has been estimated to be 20 (Garber *et al.*, 1991).

Germinal mutations of the p53 gene were first identified in several families with the Li-Fraumeni syndrome in 1990 (Malkin *et al.*, 1990). Shortly after, a number of case reports demonstrating the presence of p53 germline mutations were reported (Srivastava *et al.*, 1990; Law *et al.*, 1991; Santibanez-Koref *et al.*, 1991). Members of these family contain one mutant p53 allele and one wild-type p53 allele.

Furthermore, all the tumour-affected individuals retain the mutant allele and lose the wild-type p53 allele in their tumour tissues (Malkin *et al.*, 1990). These phenomena are consistent with the genetic changes of the RB gene in familial forms of retinoblastoma, and could well be explained by the 'two hit' hypothesis presented by Knudson (1971). It is therefore thought that the Li-Fraumeni syndrome patients are predisposed to cancer because one p53 allele is inactivated in the germline and only the remaining allele needs to be altered by somatic mutation. In normal individuals developing a sporadic tumour however, both p53 alleles must be inactivated in the same cell by somatic mutation.

The first germline mutations described were found in codons 245 and 258 within one of the evolutionary conserved domains (Malkin *et al.*, 1990). Later, the analysis of germline mutations in the Li-Fraumeni syndrome clearly indicated that they are widely distributed among the p53 gene (Srivastava *et al.*, 1990; Law *et al.*, 1991; Santibanez-Koref *et al.*, 1991). Most of the germline mutations reported so far are missense mutations located in the evolutionarily conserved domains. It should be pointed out that germline mutations for p53 have not been found in all Li-Fraumeni syndrome-like families (Santibanez-Koref *et al.*, 1991; Birch, 1992; Frebourg & Friend, 1992). These negative results must be analysed with caution. Analysis of the entire p53 gene sequence in these cases are apparently required. Alternatively, it is possible that germline p53 mutations are not the only molecular basis of the Li-Fraumeni syndrome. For example, genetic alterations affecting proteins in the p53 signalling pathway could produce phenotypes identical to the Li-Fraumeni syndrome (Barnes *et al.*, 1992; Frebourg & Friend, 1992; Oliner *et al.*, 1992).

The discovery of mutations in the tumour suppressor genes that occur not only at the somatic level but also in the germline has important clinical implications. Detection of such germline mutations should allow identification of subjects at high-risk to develop cancers (Li *et al.*, 1992). Recently, germline p53 mutations were also found in some patients who had an unusual history of cancers (e.g., multiple malignancies and a family history of cancers), but whose family histories are not fully indicative of the Li-Fraumeni syndrome (Malkin *et al.*, 1992; Sameshima *et al.*, 1992; Toguchida *et al.*, 1992). The early detection of such mutations would be useful not only in treating these patients, but also in identifying family members who may be at high-risk for the development of tumours.

### *Tumour formation in transgenic mice*

The involvement of p53 in the development of cancers was also demonstrated *in vivo* by generating transgenic mice carrying mutated p53 genomic fragments (Lavigueur *et al.*, 1989; Donehower *et al.*, 1992). By microinjection of p53 genomic fragments derived from either a Friend erythroleukaemia cell line or BALB/c mouse liver DNA into fertilised eggs of CD-1 mice, Lavigueur *et al.* (1989) generated a series of transgenic mice that express the mutant transgene in a wide variety of tissues. The widespread expression of p53 in these transgenic animals was associated with a significantly increased incidence of a broad spectrum of malignancies. Up to 20% of the transgenic mice developed neoplasms during the experiment. Although variation in the tumour types derived from different tissues were observed, lung adenocarcinomas, osteosarcomas, and lymphomas were particularly prevalent.

In the study carried out by Donehower *et al.* (1992), a null mutation was introduced into the p53 gene by homozygous recombination in murine embryonic stem cells. These cells (carrying a damaged p53 allele) were then injected into blastocysts obtained from normal mice, and the chimeric blastocysts were implanted into pseudopregnant mice. By this way, they created a unique line of transgenic mice carrying two non-functional p53 alleles. The mice were developmentally normal, however, a predisposition towards tumour formation was again noted at an early age. Nearly 75% (26/35) of the homozygotes developed a variety of tumours by 6 months (at ages from 8 to 26 weeks), and 9/26 (35%) mice had multiple neoplasms. The predominant tumour was malignant lymphoma (accounting for $>70\%$ of cases). The other tumours were sarcomas, including haemangiosarcomas, undifferentiated sarcomas, and osteosarcoma. The incidence of tumours in the homozygous mice was considerably higher than the 20% incidence observed by 18 months of age in mice carrying a mutant p53 transgene, indicating that the presence of a single wild-type p53 allele was sufficient to reduce tumour formation.

These results demonstrated that the introduction into the mouse germ line of abnormal p53 genes results in markedly elevated tumour susceptibility and provide direct evidence that p53 plays a causal role in tumour formation. The long latency period for tumour induction and the overall tumour incidence, however, suggested that alterations of the p53 gene alone may not be sufficient for tumour development and that further genetic or epigenetic changes might be required. Similar to the observations in human beings, the variety of tumours identified in the transgenic mice indicate that p53 is involved in the tumourigenesis of many tissues and cell types. The experimental results also indicate that the p53 protein plays an important role in the regulation of cell growth, but does not have an important function in the mouse development and differentiation. This in contrast to the RB tumour suppressor gene, which has been recently shown to be essential for normal mouse development (Clarke *et al.*, 1992; Jacks *et al.*, 1992; Lee *et al.*, 1992).

It was recently noted (Donehower *et al.*, 1992) that fibroblasts derived from the homozygous animals became immortalised in tissue culture much more readily than cells from either the heterozygote or wild-type mice. Thus, the loss of p53 function is closely correlated with the immortalisation of cells in tissue culture.

### *Interaction of wild-type p53 with oncoprotein of tumour viruses*

In addition to the missense mutations that cause inactivation of p53, an alternative mechanism for inactivation of the wild-type p53 is to bind to the transforming proteins of DNA tumour viruses (Levine, 1991). It has been demonstrated that the SV40 large T antigen (Tan *et al.*, 1986; Schmeig *et al.*, 1988), the adenovirus E1B protein (Sarnow *et al.*, 1982) and papillomavirus E6 protein (Scheffner *et al.*, 1990; Werness *et al.*, 1990) bind to the p53 protein (Figure 2). Similarly, the SV40 large T antigen (Decaprio *et al.*, 1988), the adenovirus E1A protein (Whyte *et al.*, 1988) and papillomavirus E7 protein (Dyson *et al.*, 1989; Münger *et al.*, 1989) bind to the RB protein (Figure 2). Binding of p53 to SV40 large T or adenovirus E1B proteins leads to an increased half-life of p53 and presumably inactivates its normal function by formation of stable complexes. In contrast, E6 proteins from papillomaviruses are known to facilitate the degradation of p53 (Scheffner *et al.*, 1990; Werness *et al.*, 1990).

Accordingly, cancers resulted from tumour virus infections may contain only wild-type p53 allele. In this regard, cervical carcinomas could be particularly instructive. Human papillomaviruses (HPVs), especially HPV types 16 and 18, have been implicated as important etiological factors in the development of human cervical cancer (Syrjänen *et al.*, 1987; Chang, 1990; Howley, 1991; zur Hausen, 1991). More than 90% of cervical carcinomas contain detectable HPV DNA sequences, many of which constantly express the E6 open reading frame (Howley, 1991). While the E6 proteins from HPV 16 and 18 bind to p53, the E6 proteins from HPV types 6 and 11 fail to bind with p53 to a detectable extent (Scheffner *et al.*, 1990; Werness *et al.*, 1990). This is of particular interest because HPV 16 and 18 are mostly associated with invasive carcinomas, and are therefore regarded as 'high-risk' HPV types, while HPV 6 or 11 are most frequently found in benign condyloma lesions (Syrjänen *et al.*, 1987; Chang, 1990; Howley, 1991; zur Hausen, 1991). Thus, the biological properties of these viruses may be correlated with their abilities to associate with the p53. Recently,

**Figure 2** Schematic diagram of the interaction between tumour virus oncoproteins, p53 and RB. The viral-encoded oncoproteins of SV40 (large T antigen), type 5 adenovirus (E1A and E1B), and human papillomavirus types 16 and 18 (E6 and E7) are indicated as rectangles. The black regions of the rectangles represent proposed sites of interaction between the viral oncoproteins and the tumour suppressor proteins. The known functions of these viral oncoproteins are indicated under the rectangles.

Mietz *et al.* (1992) demonstrated that the transcritional transactivation function of wild-type p53 could be inhibited by the SV40 large T antigen and by the HPV-16 E6 oncoprotein. Furthermore, SV40 T antigen mutants that are defective for p53 binding were not able to inhibit transactivation and, similarly, HPV E6 proteins that were either mutant or derived from non-oncogenic HPV types, also had no effect on p53 transactivation. These results suggested that the transforming functions of these viral oncoproteins may be linked to their ability to inhibit p53-mediated transcritional activation.

*Interaction of wild-type p53 with cellular gene products*

The p53 pathway may also be disrupted by alteration of a cellular gene, *MDM2* (murine double minute-2). This gene was originally identified by virtue of its amplification in a spontaneously transformed mouse cell line (Fakharzadeh *et al.*, 1991). The mouse *MDM2* gene is located in a 'mouse double minute' chromosome. This gene product was recently shown to bind to p53 (Momand *et al.*, 1992). By microsequencing, Momand *et al.* (1992) have identified a rat protein that co-immunoprecipitated with p53 as the rat homologue of the mouse *MDM2* protein. Recently, Oliner *et al.* (1992) isolated the human homologue of *MDM2*, mapped it to the long arm of human chromosome 12, and showed that it also can bind to p53. As is the case with the tumour viruses, this binding appears to inhibit the ability of p53 to transactivate genes adjacent to p53-binding sites (Momand *et al.*, 1992). The *MDM2* protein was recently found to be able to overcome the growth-suppressive properties of wild-type p53; and overexpression of the *MDM2* protein resulted in the immortalisation and transformation of primary rat embryo fibroblasts (Finlay, 1993).

It has been demonstrated that the *MDM2* gene is often amplified in human sarcomas (Oliner *et al.*, 1992). This is consistent with the previous demonstration of chromosomal aberrations at this site in these tumours. Therefore, high numbers of *MDM2* gene copies and the consequent overexpression of the *MDM2* protein may, like tumour virus oncoproteins, inactivate normal p53 functions by complexing to it.

**Mechanisms involved in p53 inactivation**

The reasons for tissue-specific mutations of the p53 gene are unknown. These differences may reflect variable constraints imposed by the biological characteristics of different tissues or exposure to different types of carcinogens (Cohen & Ellwein, 1991; Harris, 1991; Hollstein *et al.*, 1991; Strauss, 1992). Differences in metabolic and DNA repair capacities in different tissues and cell types are some of the factors expected to underline these differences. Typical base changes in characteristic locations can be related to molecular mechanisms such as covalent binding of DNA bases by electrophilic carcinogen metabolites. Endogenous mechanisms, e.g. DNA polymerase infidelity, depurination, oxidative damage from free radicals generated by biological processes, and deamination of 5-methylcytosine can also lead to mutations (Harris, 1991; Hollstein *et al.*, 1991; Strauss, 1992).

Of the known exogenous carcinogens, many elicit base substitutions in bacteria, mammalian cells *in vitro* and in experimental animals (Harris, 1991; Strauss, 1992). This activity has been used in short-term tests to identify candidate carcinogens. Electrophilic attack of DNA bases by carcinogen metabolites, followed by fixation of the damage at the site of the adducted base during DNA replication has been the principal model of action. Ample biochemical data exist on the adducts and specific base substitutions expected from electrophilic metabolites of various important classes of carcinogens, including *N*-nitrosamines, polycyclic aromatic hydrocarbons, and fungal toxins, being consistent with the results of animal experiments on the specificity of tumour mutations in *ras*. The mechanisms of p53 inactivation due to the formation of protein complexes between viral oncoprotein or cellular gene product and wild-type p53 protein, differ considerably from those elicited by gene alterations. Tumours resulted from this pathway may contain only wildtype p53. Notable examples include cervical carcinomas associated with HPV infection, and sarcomas with *MDM2* amplification. In both cervical carcinoma (Crook *et al.*, 1991; Scheffner *et al.*, 1991) and sarcomas with *MDM2* amplification (Oliner *et al.*, 1992), p53 mutations appear to be rare, whereas such mutations are common in other anogenital malignancies which are not associated with tumour virus infections, and sarcomas without *MDM2* amplification.

### A model for p53-associated tumourigenesis

The molecular mechanisms by which the p53 is involved in tumourigenesis are not fully understood. Based on recent data, Vogelstein and Kinzler (1992) proposed a model for p53 gene and gene product function in cell growth control. In this model, the wild-type p53 gene binds to p53-binding sites as a tetramer and stimulates the expression of downstream genes that negatively control cell growth (Stenger *et al.*, 1992; Vogelstein & Kinzler, 1992). Under normal conditions, the wild-type p53 appears to play little part in cell cycle control. However, when cells or cellular DNA are damaged, the cells increase p53 expression. The accumulation of wild-type p53 switches off the cell cycle until the damage is repaired. If the repair fails, p53 may further trigger controlled cell death through apoptosis (Lane, 1992; Vogelstein & Kinzler, 1992). On the other hand, cells with p53 allelic losses or with mutant p53 genes are only partially blocked, and therefore acquire a selective growth advantage (vogelstein & Kinzler, 1992). Clonal expansion of these cells, in combination with the inactivation of other tumour suppressor genes and/or activation of certain oncogenes as well as other genetic changes in the cell, may lead to tumour formation and progression. Consistent with this hypothesis, recent results show that loss of wild-type p53 leads to gene amplification at high frequency and enhances the possibility of genomic rearrangements (Livingstone *et al.*, 1992; Yin *et al.*, 1992). The enhanced genomic instability may, therefore, accelerate the occurrence of certain genetic changes that permit cells to overcome the normal strictures against excessive multiplication and metastasis and lead to tumour formation and progression.

As shown in Figure 3, loss of normal p53 function could be achieved in a variety of ways, including genetic changes in the p53 gene (e.g., germline mutations, somatic mutation, small or large deletions, structural rearrangements, and genomic insertions), formation of protein complexes with viral oncoproteins (e.g., the SV40 T antigen, adenovirus E1B, and papillomavirus E6), and binding to the cellular gene products (e.g., *MDM2*). Therefore, analysis of the p53 gene and gene product in tumour cells may reveal significant differences in its involvement between stages of tumorigenesis, tumour types as well as their tissues of origin (Vogelstein & Kinzler, 1992). In some tumours, a loss of one or both p53 alleles may occur, which reduces the concentration of p53 tetramers below that required to perform their normal function. In other tumours, a nonsense mutation results in the truncation of p53. More commonly, one allele of p53 develops a missense mutation, and this is accompanied by a deletion of the other allele, resulting in the absence of any wild-type p53 tetramers. This occurs in many tumours, including those of the colon, brain, lung, liver, esophagus, and bladder. In cervical cancers, the expression of HPV E6 results in the functional inactivation of the p53 through binding and degradation. In soft-tissue sarcoma, the amplification of *MDM2* and the binding of its products to p53 creates a similar loss of functional p53.

These data support the notion that p53 is a common cellular target in human carcinogenesis mediated by endogenous factors and exogenous carcinogens as well as tumour viruses.

**Figure 3** Schematic representation of the p53 as a common cellular target in tumorigenesis. Inactivation of the wild-type p53 can be achieved in a variety of ways, e.g., exposure to physical factors and chemical carcinogens, infections with tumour viruses, germline mutations, and binding to the specific cellular gene product.

**Conclusions and future prospects**

The identification of tumour suppressor genes has broadened our view of the molecular basis of carcinogenesis. However, many key issues on the role of p53 gene function and its association with human carcinogenesis still remain to be clarified. Further studies are needed to elucidate the role of p53 in normal and tumour cells. We need to know more about the frequency of p53 mutations in human tumours. The investigation of the cell and molecular biology of the p53 in tumours should provide further insights into the regulation of cell proliferation and its disturbance in tumour cells. With the advances in our understanding of the process of malignant transformation associated with p53 gene alterations, some practical applications, e.g., the early detection of premalignancies and malignancies by molecular diagnosis as well as the possibility of gene therapy, may emerge.

The original studies of the author (F.C.) has been supported by a research grant from the Savo Cancer Fund. The excellent help of Mrs Wang Lijuan in preparation of the manuscript is highly appreciated.

**References**

ALLRED, D.C., CLARK, G.M., ELLEDGE, R., FUQUA, S.A.W., BROWN, R.W., CHAMNESS, G.C., OSBORNE, C.K. & MCGUIRE, W.L. (1993). Association of p53 protein expression with tumor cell proliferation rate and clinical outcome in node-negative breast cancer. *J. Natl Cancer Inst.*, **85,** 200–206.

BAKER, S.J., FEARON, E.R., NIGRO, J.M., HAMILTON, S.R., PREISINGER, A.C., JESSUP, J.M., VAN TUINEN, P., LEDBETTER, D.H., BARKER, D.F., NAKAMURA, Y., WHITE, R. & VOGELSTEIN, B. (1989). Chromosome 17 deletions and p53 gene mutations in colorectal carcinoma. *Science*, **244,** 217–221.

BAKER, S.J., MARKOWITZ, S., FEARON, E.R., WILLSON, J.K.V. & VOGELSTEIN, B. (1990). Suppression of human colorectal carcinoma cell growth by wild-type p53. *Science*, **249,** 912–915.

BARNES, D.M., HANBY, A.M., GILLETT, C.E., MOHAMMED, E., HODGSON, S., BOBROW, L.G., LEIGH, I.M., PURKIS, T., MACGEOCH, C., SPURR, N.K., BARTEK, J., VOJTESEK, B., PICKSLEY, S.M. & LANE, D.P. (1992). Abnormal expression of wild type p53 protein in normal cells of a cancer family patient. *Lancet*, **340,** 259–263.

BARTEK, J., BARTKOVA, J., VOJTESEK, B., STASKOVA, Z., LUKAS, J., REJTHAR, A., KOVARIK, J., MIDGLEY, C.A., GANNON, J.V. & LANE, D.P. (1991). Aberrant expression of the p53 oncoprotein is a common feature of a wide spectrum of human malignancies. *Oncogene*, **6,** 1699–1703.

BELL, S.M., SCOTT, N., CROSS, D., SAGAR, P., LEWIS, F.A., BLAIR, G.E., TAYLOR, G.R., DIXON, M.F. & QUIRKE, P. (1993). Prognostic value of p53 overexpression and c-Ki-ras gene mutations in colorectal cancer. *Gastroenterology*, **104,** 57–64.

BENNETT, W.P., HOLLSTEIN, M.C., METCALF, R.A., WELSH, J.A., HE, A., ZHU, S.M., KUSTERS, I., RESAU, J.H., TRUMP, B.F., LANE, D.P. & HARRIS, C.C. (1992). p53 mutation and protein accumulation during multistage human esophageal carcinogenesis. *Cancer Res.*, **52,** 6092–6097.

BIRCH, J.M. (1992). Germline mutations in the p53 tumour suppressor gene: scientific, clinical and ethical challenges. *Br. J. Cancer*, **66,** 424–426.

BISHOP, J.M. (1987). The molecular genetics of cancers. *Science*, **235,** 305–311.

BOS, J.L. (1989). *ras* oncogenes in human cancer: a review. *Cancer Res.*, **49,** 4682–4689.

BRASH, D.E., RUDOLPH, J.A., SIMON, J.A., LIN, A., MCKENNA, G.J., BADEN, H.P., HALPERIN, A.J. & PONTEN, J. (1991). A role for sunlight in skin cancer: UV-induced p53 mutations in squamous cell carcinoma. *Proc. Natl Acad. Sci. USA*, **88,** 10120–10128.

BRESSAC, B., KEW, M., WANDS, J. & OZTURK, M. (1991). Selective G to T mutations of p53 gene in hepatocellular carcinoma from southern Africa. *Nature*, **350,** 429–431.

CANTLEY, L.C., AUGER, K.R., CARPENTER, C., DUCKWORTH, B., GRAZIANI, A., KAPELLER, R. & SOLTOFF, S. (1991). Oncogenes and signal transduction. *Cell*, **64,** 281–302.

CHANG, F. (1990). Role of papillomaviruses. *J. Clin. Pathol.*, **43,** 269–276.

CHANG, F., SYRJÄNEN, S., KURVINEN, K. & SYRJÄNEN, K. (1993). The p53 tumor suppressor gene as a common cellular target in human carcinogenesis. *Am. J. Gastroenterol.*, **88,** 174–186.

CHEN, P.L., CHEN, Y.M., BOOKSTEIN, R. & LEE, W.H. (1990). Genetic mechanisms of tumor suppression by the human p53 gene. *Science*, **250,** 1576–1580.

CALLAHAN, R., CROPP, C.S., MERLO, G.R., LISCIA, D.S., CAPPA, A.P. & LIDEREAU, R. (1992). Somatic mutations and human breast cancer. A status report. *Cancer*, **69**(Suppl.), 1582–1588.

CLARKE, A.R., MAANDAG, E.R., VAN ROON, M., VAN DER LUGT, N.M.T., VAN DER VALK, M., HOOPER, M.L., BERN, A. & TE RIELE, H. (1992). Requirement for a functional Rb-1 gene in murine development. *Nature*, **359,** 328–330.

COHEN, S.M. & ELLWEIN, L.B. (1991). Genetic errors, cell proliferation, and carcinogenesis. *Cancer Res.*, **51,** 6493–6505.

CROOK, T., WREDE, D. & VOUSDEN, K.H. (1991). p53 point mutation in HPV negative human cervical carcinoma cell lines. *Oncogene*, **6,** 873–875.

DECAPRIO, J.A., LUDLOW, J.W., FRIGGE, J., SHEW, J.-Y., HUANG, C.-M., LEE, W.-H., MARSILLO, E., PAUCHA, E. & LIVINGSTONE, D.M. (1988). SV40 large tumor anitgen forms a specific complex with the product of the retinoblastoma susceptibility gene. *Cell*, **54,** 275–283.

DILLER, L., KASSEL, J., NELSON, C.E., GRYKA, M.A., LITWAK, G., GEBHARDT, M., BRESSAC, B., OZTURK, M., BAKER, S.J., VOGELSTEIN, B. & FRIEND, S.H. (1990). p53 function as a cell cycle control protein in osteosarcomas. *Mol. Cell. Biol.*, **10,** 5772–5781.

DONEHOWER, L.A., HARVEY, M., SLAGLE, B.L., MCARTHUR, M.J., MONTGOMERY, C.A., Jr, BUTEL, J.S. & BRADLEY, A. (1992). Mice deficient for p53 are developmentally normal but susceptible to spontaneous tumours. *Nature*, **356,** 215–221.

DYSON, N., HOWLEY, P.M., MÜNGER, K. & HARLOW, E. (1989). The human papillomavirus-16 E7 oncoprotein is able to bind to the retinoblastoma gene product. *Science*, **243,** 934–936.

EFFERT, P., MCCOY, R., ABDEL-HAMID, M., FLYNN, K., ZHANG, Q., BUSSON, P., TURSZ, T., LIU, E. & RAAB-TRAUB, N. (1992). Alterations of the p53 gene in nasopharyngeal carcinoma. *J. Virol.*, **66,** 3768–3775.

ELIYAHU, D., MICHALOVITZ, D., ELIYAHU, S., PINHASI-KIMHI, O. & OREN, M. (1989). Wild-type p53 can inhibit oncogene-mediated focus formation. *Proc. Natl Acad. Sci. USA*, **86,** 8763–8767.

FAKHARZADEH, S.S., TRUSKO, S.P. & GEORGE, D.L. (1991). Tumorigenic potential associated with enhanced expression of a gene that is amplified in mouse tumor cell line. *EMBO J.*, **10,** 1565–1569.

FARMER, G., BARGONETTI, J., ZHU, H., FRIEDMAN, P., PRYWES, R. & PRIVES, C. (1992). Wild-type p53 activates transcription *in vitro*. *Nature*, **358,** 83–86.

FEARON, E.R. & VOGELSTEIN, B. (1990). A genetic model for colorectal tumorigenesis. *Cell*, **61,** 759–767.

FINLAY, C.A., HINDS, P.W. & LEVINE, A.J. (1989). The p53 proto-oncogene can act as a suppressor of transformation. *Cell*, **57,** 1083–1093.

FINLAY, C.A. (1993). The mdm-2 oncogene can overcome wild-type p53 suppression of transformed cell growth. *Mol. Cell. Biol.*, **13,** 301–306.

FREBOURG, T. & FRIEND, S.H. (1992). Cancer risks from germline p53 mutations. *J. Clin. Invest.*, **90,** 1637–1641.

FRIEDMAN, P., KERN, S., VOGELSTEIN, B. & PRIVES, C. (1990). Wild-type, but not mutant human p53 proteins inhibit the replication activities of simian virus 40 large tumor antigen. *Proc. Natl Acad. Sci. USA*, **87,** 9275–9279.

FULTS, D., BROCKMEYER, D., TULLOUS, M.W., PEDONE, C.A. & CAWTHON, R.M. (1992). p53 mutation and loss of heterozygosity on chromosomes 17 and 10 during human astrocytoma progression. *Cancer Res.*, **52,** 674–679.

GARBER, J.E., GOLDSTEIN, A.M., KANTOR, A.F., DREYFUS, M.G., FRAUMENI, J.F. & LI, F.P. (1991). Follow-up study of twenty-four families with Li-Fraumeni syndrome. *Cancer Res.*, **51,** 6094–6097.

GUSTERSON, B.A., ANBAZHAGAN, R., WARREN, W., MIDGELY, C., LANE, D.P., O'HARE, M., STAMPS, A., CARTER, R. & JAYATILAKE, H. (1991). Expression of p53 in premalignant and malignant squamous epithelium. *Oncogene*, **6,** 1785–1789.

HALL, P.A., MCKEE, P.H., MENAGE, H.P., DOVER, R. & LANE, D.P. (1993). High levels of p53 protein in UV-irradiated normal human skin. *Oncogene*, **8,** 203–207.

HARRIS, C.C. (1991). Chemical and physical carcinogenesis: advances and perspectives for the 1990s. *Cancer Res.*, **51** (Suppl.), 5023s–5044s.

HARTWELL, L. (1992). Defects in a cell cycle checkpoint may be responsible for the genomic instability of cancer. *Cell*, **71**, 543–546.

HINDS, P., FINLAY, C. & LEVINE, A.J. (1989). Mutation is required to activate the p53 gene for cooperation with the *ras* oncogene and transformation. *J. Virol.*, **63**, 739–746.

HOLLSTEIN, M.C., PERI, L., MANDARD, A.M., WELSH, J.A., MONTESANO, R., METCALF, R.A., BAK, M. & HARRIS, C.C. (1991). Genetic analysis of human esophageal tumors from two high incidence geographic areas: frequent p53 base substitutions and absence of *ras* mutations. *Cancer Res.*, **51**, 4102–4106.

HOLLSTEIN, M., SIDRANSKY, D., VOGELSTEIN, B. & HARRIS, C.C. (1991). p53 mutations in human cancers. *Science*, **253**, 49–53.

HOWLEY, P.M. (1991). Role of the human papillomaviruses in human cancer. *Cancer Res.*, **51**, 5019–5022.

HSIA, C.C., KLEINER, D.E. Jr, AXIOTIS, C.A., DI BISCEGLIE, A., NOMURA, A.M.Y., STEMMERMANN, G.N. & TABOR, E. (1992). Mutations of p53 in hepatocellular carcinoma: roles of hepatitis B virus and aflatoxin contamination in the diet. *J. Natl Cancer Inst.*, **84**, 1638–1641.

HSU, I.C., METCALF, R.A., SUN, T., WELSH, J.A., WANG, N.J. & HARRIS, C.C. (1991). Mutational hotspot in the p53 gene in human hepatocellular carcinomas. *Nature*, **350**, 427–428.

HUPP, T.R., MEEK, D.W., MIDGLEY, C.A. & LANE, D.P. (1992). Regulation of the specific DNA binding function of p53. *Cell*, **71**, 875–886.

ICHIKAWA, A., HOTTA, T., TAKAGI, N., TSUSHITA, K., KINOSHITA, T., NAGAI, H., MURAKAMI, Y., HAYASHI, K. & SAITO, H. (1992). Mutations of p53 gene and their relation to disease progression in B-cell lymphoma. *Blood*, **79**, 2701–2707.

JACKS, T., FAZELI, A., SCHMITT, E.M., BRONSON, R.T., GOODELL, M.A. & WEINBERG, R.A. (1992). Effects of an Rb mutation in the mouse. *Nature*, **359**, 295–300.

JENKINS, J.R., RUDGE, K. & CURRIE, G.A. (1984). Cellular immortalization by a cDNA clone encoding the transformation-associated phosphoprotein p53. *Nature*, **312**, 651–654.

KAKEJI, Y., KORENAGA, D., TSUJITANI, S., BABA, H., ANAI, H., MAEHARA, Y. & SUGIMACHI, K. (1993). Gastric cancer with p53 overexpression has high potential for metastasising to lymph nodes source. *Br. J. Cancer*, **67**, 589–593.

KASTAN, M.B., ZHAN, Q.M., ELDEIRY, W.S., CARRIER, F., JACKS, T., WALSH, W.V., PLUNKETT, B.S., VOGELSTEIN, B. & FORNACE, A.J. (1992). A mammalian cell cycle checkpoint pathway utilizing p53 and GADD45 is defective in Ataxia-Telangiectasia. *Cell*, **71**, 587–597.

KERN, S.E., KINZLER, K., BRUSKIN, A., JAROSZ, D., FRIEDMAN, P., PRIVES, C. & VOGELSTEIN, B. (1991). Identification of p53 as a sequence specific DNA binding protein. *Science*, **252**, 1708–1711.

KERN, S.E., PIETENPOL, J.A., THIAGALINGAM, S., SEYMOUR, A., KINZLER, K.W. & VOGELSTEIN, B. (1992). Oncogenic forms of p53 inhibit p53-regulated gene expression. *Science*, **256**, 827–830.

KNUDSON, A.G., JR, (1971). Mutation and cancer. Statistical analysis of retinoblastoma. *Proc. Natl Acad. Sci. USA*, **68**, 820–823.

KRESS, S., SUTTER, C., STRICKLAND, P.T., MUKHTAR, H., SCHWEIZER, J. & SCHWARZ, M. (1992). Carcinogen-specific mutational pattern in the p53 gene in ultraviolet B radiation-induced squamous cell carcinomas of mouse skin. *Cancer Res.*, **52**, 6400–6403.

LANE, D.P. & CRAWFORD, L.V. (1979). T antigen is bound to a host protein in SV40-transformed cells. *Nature*, **278**, 261–263.

LANE, D.P. & BENCHIMOL, S. (1990). p53: oncogene or anti-oncogene? *Gene Develop.*, **4**, 1–8.

LANE, D.P. (1992). p53, guardian of the genome. *Nature*, **358**, 15–16.

LAVIGUEUR, A., MALTBY, V., MOCK, D., ROSSANT, J., PAWSON, T. & BERNSTEIN, A. (1989). High incidence of lung, bone, and lymphoid tumors in transgenic mice overexpressing mutant alleles of the p53 oncogene. *Mol. Cell. Biol.*, **9**, 3982–3991.

LAW, J.C., STRONG, L.C., CHIDAMBARAM, A. & FERRELL, R.E. (1991). A germ line mutation in exon 5 of the p53 gene in an extended cancer family. *Cancer Res.*, **51**, 6385–6387.

LEE, E.Y.H.P., CHANG, C.Y., HU, N., WANG, Y.C.J., LAI, C.C., HERRUP, K., LEE, W.H. & BRADLEY, A. (1992). Mice deficient for Rb are nonviable and show defects in neurogenesis and haematopoiesis. *Nature*, **359**, 288–294.

LEVINE, A.J. (1990). The p53 protein and its interactions with the oncogene products of the small DNA tumor viruses. *Virology*, **177**, 419–426.

LEVINE, A.J., MOMAND, J. & FINLAY, C.A. (1991). The p53 tumour suppressor gene. *Nature*, **351**, 453–456.

LI, F.P., FRAUMENI, J.F. Jr, MULVIHILL, J.J., BLATTNER, W.A., DREYFUS, M.G., TUCKER, M.A. & MILLER, R.W. (1988). A cancer family syndrome in twenty four kindreds. *Cancer Res.*, **48**, 5358–5362.

LI, F.P., GARBER, J.E., FRIEND, S.H., STRONG, L.C., PATENAUDE, A.F., JUENGST, E.T., REILLY, P.R., CORREA, P. & FRAUMENI, J.F. Jr. (1992). Recommendations on predictive testing for germ line p53 mutations among cancer-prone individuals. *J. Natl Cancer Inst.*, **84**, 1156–1160.

LINZER, D.I.H. & LEVINE, A.J. (1979). Characterization of a 54K dalton cellular SV40 tumor antigen present in SV40 transformed cells and uninfected embryonal carcinoma cells. *Cell*, **17**, 43–52.

LIVINGSTONE, L.R., WHITE, A., SPROUSE, J., LIVANOS, E., JACKS, T. & TISTY, T.D. (1992). Altered cell cycle arrest and gene amplification potential accompany loss of wild-type p53. *Cell*, **70**, 923–935.

LU, X., PARK, S.H., THOMPSON, T.C. & LANE, D.P. (1993). *ras*-induced hyperplasia occurs with mutation of p53, but activated *ras* and *myc* together can induce carcinoma without p53 mutation. *Cell*, **70**, 153–161.

MALKIN, D., LI, F.P., STRONG, L.C., FRAUMENI, J.F. Jr, NELSON, C.E., KIM, D.H., KASSEL, J., GRYKA, M.A., BISCHOFF, F.Z., TAINSKY, M.A. & FRIEND, S.H. (1990). Germ line p53 mutations in a familial syndrome of breast cancer, sarcomas, and other neoplasms. *Science*, **250**, 1233–1238.

MALKIN, D., JOLLY, K.W., BARBIER, N., LOOK, T., FRIEND, S.H., GEBHARDT, M.C., ANDERSEN, T.I., BØRRESEN, A.L., LI, F.P., GARBER, J. & STRONG, L.C. (1992). Germline mutations of the p53 tumor-suppressor gene in children and young adults with second malignant neoplasms. *N. Engl. J. Med.*, **326**, 1309–1315.

MARSHALL, C.J. (1991). Tumor suppressor genes. *Cell*, **64**, 313–326.

MAZARS, R., SPINARDI, L., BECHEIKH, M., SIMONY-LAFONTAINE, J., JEANTEUR, P. & THEILLET, C. (1992). p52 mutations occur in aggressive breast cancer. *Cancer Res.*, **52**, 3918–3923.

MCBRIDE, O.W., MERRY, D. & GIVOL, D. (1986). The gene for human p53 cellular tumor antigen is located on chromosome 17 short arm (17p13). *Proc. Natl Acad. Sci. USA*, **83**, 130–134.

MERCER, W.E., SHIELDS, M.T., AMIN, M., SAUVE, G.J., APPELLA, E., ROMANO, J.W. & ULLRICH, S.J. (1990). Negative growth regulation in a glioblastoma tumor cell line that conditionally expresses human wild-type p53. *Proc. Natl Acad. Sci. USA*, **87**, 6166–6170.

MIETZ, J.A., UNGER, T., HUIBREGTSE, J.M. & HOWLEY, P.M. (1992). The transcritional transactivation function of wild-type-p53 is inhibited by SV40 large T-antigen and by HPV-16 E6-oncoprotein. *EMBO J.*, **11**, 5013–5020.

MILLER, C., MOHANDAS, T., WOLF, D., PROKOCIMER, M., ROTTER, V. & KOEFFLER, P.H. (1986). Human p53 localized to short arm of chromosome 17. *Nature*, **319**, 783–784.

MILLER, C.W., SIMON, K., ASLO, A., KOK, K., YOKOTA, J., BUYS, C.H., TERADA, M. & KOEFFLER, H.P. (1992). p53 mutations in human lung tumors. *Cancer Res.*, **52**, 1695–1698.

MOMAND, J., ZAMBETTI, G.P., OLSON, D.C., GEORGE, D. & LEVINE, A.J. (1992). The *mdm-2* oncogene product forms a complex with the p53 protein and inhibits p53-mediated transactivation. *Cell*, **69**, 1237–1245.

MÜNGER, K., WERNESS, B.A., DYSON, N., PHELPS, W.C. & HOWLEY, P.M. (1989). Complex formation of human papillomavirus E7 proteins with the retinoblastoma tumor suppressor gene product. *EMBO J.*, **8**, 4099–4109.

NIGRO, J.M., BAKER, S.J., PREISINGER, A.C., JESSUP, J.M., HOSTETTER, R., CLEARY, K., BIGNER, S.H., DAVIDSON, N., BAYLIN, S., DEVILEE, P., GLOVER, T., COLLINS, F.S., WESTON, A., MODALI, R., HARRIS, C.C. & VOGELSTEIN, B. (1989). Mutations in the p53 gene occur in diverse human tumour types. *Nature*, **342**, 705–708.

OLINER, J.D., KINZLER, K.W., MELTZER, P.S., GEORGE, D.L & VOGELSTEIN, B. (1992). Amplification of a gene encoding a p53-associated protein in human sarcomas. *Nature*, **358**, 80–83.

PARADA, L.F., LAND, H., WEINBERG, R.A., WOLF, D. & ROTTER, V. (1984). Cooperation between gene encoding p53 tumour antigen and *ras* in cellular transformation. *Nature*, **312**, 649–651.

PORTER, P.L., GOWN, A.M., KRAMP, S.G. & COLTRERA, M.D. (1992). Widespread p53 overexpression in human malignant tumors: an immunohistochemical study using methacarn-fixed, embedded tissue. *Am. J. Pathol.*, **140**, 145–153.

PUISIEUX, A., LIM, S., GROOPMAN, J. & OZTURK, M. (1991). Selective targeting of p53 gene mutational hotspots in human cancers by eitological defined carcinogens. *Cancer Res.*, **51**, 6185–6189.

ROVINSKI, B. & BENCHIMOL, S. (1988). Immortalization of rat embryo fibroblasts by the cellular p53 oncogene. *Oncogene*, **2**, 445–452.

SAMESHIMA, Y., TSUNEMATSU, Y., WATANABE, S., TSUKAMOTO, T., KAWA-HA, K., HIRATA, Y., MIZOGUCHI, H., SUGIMURA, T., TERADA, M. & YOKOTA, J. (1992). Detection of novel germ-line p53 mutations in diverse-cancer-prone families identified by selecting patients with childhood adrenocortical carcinoma. *J. Natl Cancer Inst.*, **84**, 703–707.

SANTIBANEZ-KOREF, M.F., BIRCH, J.M., HARTLEY, A.L., MORRIS JONES, P.H., CRAFT, A.W., EDEN, T., CROWTHER, D., KELSEY, A.M. & HARRIS, M. (1991). p53 germline mutations in Li-Fraumeni syndrome. *Lancet*, **338**, 1490–1491.

SARNOW, P., HO, Y.S., WILLIAMS, J. & LEVINE, A.J. (1982). Adenovirus E1b-58kd tumor antigen and SV40 large tumor antigen are physically associated with the same 54kd cellular protein in transformed cells. *Cell*, **28**, 387–394.

SCHEFFNER, M., WERNESS, B.A., HUIBREGTSE, J.M., LEVINE, A.J. & HOWLEY, P.M. (1990). The E6 oncoprotein encoded by human papillomavirus types 16 and 18 promotes the degeneration of p53. *Cell*, **63**, 1129–1136.

SCHEFFNER, M., MÜNGER, K., BYRNE, J.C. & HOWELY, P.M. (1991). The state of the p53 and retinoblastoma genes in human cervical cell lines. *Proc. Natl Acad. Sci. USA*, **88**, 5523–5527.

SCHMEIG, F.I. & SIMMONS, D.T. (1988). Characterization of the *in vitro* interaction between SV40 T antigen and p53: Mapping the p53 binding site. *Virology*, **164**, 132–140.

SHAW, P., TARDY, S., BENITO, E., OBRADOR, A. & COSTA, J. (1991). Occurrence of Ki-*ras* and p53 mutations in primary colorectal tumors. *Oncogene*, **6**, 2121–2128.

SOUSSI, T., CARON DE FROMENTEL, C. & MAY, P. (1990). Structural aspects of the p53 protein in relation to gene evolution. *Oncogene*, **5**, 945–952.

SRIVASTAVA, S., ZOU, Z.Q., PIROLLO, K., BLATTNER, W. & CHANG, E.H. (1990). Germ-line transmission of a mutated p53 gene in a cancer-prone family with Li-Fraumeni syndrome. *Nature*, **348**, 747–749.

STENGER, J.E., MAYR, G.A., MANN, K. & TEGTMEYER, P. (1992). Formation of stable p53 homotetramers and multiples of tetramers. *Mol. Carcinog.*, **5**, 102–106.

STRAUSS, B.S. (1992). The origin of point mutations in human tumor cells. *Cancer Res.*, **52**, 249–253.

SUGIMOTO, K., TOYOSHIMA, H., SAKAI, R., MIYAGAWA, K., HAGIWARA, K., ISHIKAWA, F., TAKAKU, F., YAZAKI, Y. & HIRAI, H. (1992). Frequent mutations in the p53 gene in human myeloid leukemia cell lines. *Blood*, **79**, 2378–2383.

SYRJÄNEN, K., GISSMANN, L. & KOSS, L.G. (1987). *Papillomaviruses and Human Disease*. Springer-Verlag: Heidelberg.

TAKAHASHI, T., NAU, M.M., CHIBA, I., BIRRER, M.J., ROSENBERG, R.K., VINOCOUR, M., LEVITT, M., PASS, H., GAZDAR, A.F. & MINNA, J.D. (1989). p53: a frequent target for genetic abnormalities in lung cancer. *Science*, **246**, 491–494.

TAMURA, G., KIHANA, T., NOMURA, K., TERADA, M., SUGIMURA, T. & HIROHASHI, S. (1991). Detection of frequent p53 gene mutations in primary gastric cancer by cell sorting and polymerase chain reaction single-strand conformation polymorphism analysis. *Cancer Res.*, **51**, 3056–3058.

TAN, T.-H., WALLIS, J. & LEVINE, A.J. (1986). Identification of the p53 protein domain involved in the formation of the SV40 large T antigen p53 protein complex. *J. Virol.*, **59**, 574–583.

TOGUCHIDA, J., YAMAGUCHI, T., DAYTON, S.H., BEAUCHAMP, R.L., HERRERA, G.E., ISHIZAKI, K., YAMAMURO, T., MEYERS, P.A., LITTLE, J.B., SASAKI, M., WEICHSELBAUM, R.R. & YANDELL, D.W. (1992). Prevalence and spectrum of germline mutations of the p53 gene among patients with sarcoma. *N. Engl. J. Med.*, **326**, 1301–1308.

VOGELSTEIN, B. & KINZLER, K.W. (1992). p53 function and dysfunction. *Cell*, **70**, 523–526.

WEINBERG, R.A. (1991). Tumor suppressor genes. *Science*, **254**, 1138–1146.

WERNESS, B.A., LEVINE, A.J. & HOWLEY, P.M. (1990). The E6 proteins encoded by human papillomavirus types 16 and 18 can complex p53 *in vitro*. *Science*, **248**, 76–79.

WHYTE, P., BUCHKOVICH, K.J., HOROWITZ, J.M., FRIEND, S.H., RAYBUCK, M., WEINBERG, R.A. & HARLOW, E. (1988). Association between an oncogene and an anti-oncogene; the adenovirus E1a proteins bind to the retinoblastoma gene product. *Nature*, **334**, 124–129.

WRIGHT, P.A., LEMOINE, N.R., GORETZKY, P.E., WYLLIE, F.S., BOND, J., HUGUES, C., RÖHER, H.D., WILLIAMS, D.E. & WYNFORD-THOMAS, D. (1991). Mutation of the p53 gene in a differentiated human thyroid carcinoma cell line, but not in primary thyroid tumours. *Oncogene*, **6**, 1693–1699.

WYNFORD-THOMAS, D. (1992). p53 in tumour pathology: can we trust immunocytochemistry? *J. Pathol.*, **166**, 329–330.

YIN, Y., TAINSKY, M.A., BISCHOFF, F.Z., STRONG, L.C. & WAHL, G.M. (1992). Wild-type p53 restores cell cycle control and inhibits gene amplification in cells with mutant p53 alleles. *Cell*, **70**, 937–948.

YONISH-ROUACH, E., RESNITZKY, D., LOTEM, J., SACHS, L., KIMCHI, A. & OREN, M. (1991). Wild-type p53 induces apoptosis of myeloid leukaemic cells that is inhibited by interleukin 6. *Nature*, **352**, 345–347.

ZUR HAUSEN, H. (1991). Viruses in human cancers. *Science*, **254**, 1167–1173.